# Extraction-free analysis in cosmetics by digital image colorimetry, illustrated by the quantification of urea

**DOI:** 10.1016/j.heliyon.2024.e25503

**Published:** 2024-02-01

**Authors:** Georgia Eleni Tsotsou

**Affiliations:** a Laboratory of Chemistry, Biochemistry and Cosmetology, Department of Biomedical Sciences, University of West Attica, Egaleo, 122 43, Greece; b R&D Department, COSMETIC, Ioannou Metaxa 56, Karellas, Koropi, 19400, Greece

**Keywords:** Emulsion analysis, Urea assay, Smartphone camera, Extraction-free analytical method, Digital image colorimetry

## Abstract

An extraction-free methodology is proposed for quantifying urea in cosmetics, which relies on urea-mediated decrease of methyl red decoloration by sodium hypochlorite. The method is applied directly to the cosmetic formulation and the resulting color intensity is captured by a smartphone camera. We demonstrate a linear relationship between color intensity and urea concentration in O/W emulsions and a shampoo. This quantification methodology is fully validated by determining its technical characteristics in an O/W cosmetic emulsion: The standard curve is linear over 2.5–30.0 % w/w urea (R^2^ ≥ 0.985). The coefficient of variation (CV %) on all quality control levels is ≤ 12.54 % for intermediate precision, indicating acceptable precision. Bias is up to ±4.93 % in the emulsion, indicating acceptable accuracy and a countable matrix effect. The proposed analysis setup in combination with a standard addition methodology is applied to verify urea content in purpose-made emulsions: bias is ≤±10.9 %, even in the presence of interfering ammonia. We finally demonstrate that the camera-captured color intensity of an O/W emulsion is proportional to different colorant concentrations in the formulation. This opens the route for further applications of the proposed setup to other ingredients capable of generating a colored product upon suitable reaction inside the formulation matrix.

## Highlights

• Extraction-free camera-based analysis in cosmetic O/W emulsions and shampoos.
• Linear relationship between emulsion urea levels and digital image color intensity.
• Acceptable technical characteristics for urea quantification in O/W emulsion.
• Acceptable bias upon urea quantification within cosmetic quality control.

## Graphical abstract

An Extraction-free, smartphone camera-based method for urea quantification directly in O/W emulsions and shampoos has been developed and validated.

**Figure:**
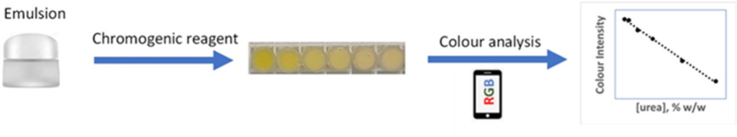

## 1 Introduction

Urea is a hygroscopic molecule, component of the natural moisturizing factor (NMF), and is often used in skin care applications. It is commonly present in moisturiser formulations at concentrations of 1–10 % w/w to achieve skin hydration and treat dry skin-associated medical conditions like atopic dermatitis, ichthyosis, xerosis and contact eczema [1]. The application of urea-containing moisturizing formulations restores the cutaneous barrier reducing skin roughness and peeling [2]. Urea may also enhance skin penetration of compounds present in the formulation and therefore transdermal application of drugs [2,3]. A review study has suggested that topical urea, as an adjunct to topical and oral antifungal treatment regimens, may improve the efficacy of onychomycosis treatment [4]. Higher than 10 % urea concentrations can denature proteins such as keratin and are, for this reason, used in keratolytic formulations. Even higher urea concentrations (≥30 %) in conjunction with papain are used for necrotic tissue debridement [5].

The Kjeldahl method is the official method for determining urea in cosmetics and dermatological formulations, which suffers mainly from poor selectivity and long pre-treatment and procedure times [6]. A number of alternative methods have been published for quality control purposes in the dermatological and cosmetic industry, including spectrophotometry [7,8], electrochemistry [9], column chromatography with ultraviolet detection [10,11], HPTLC combined with densitometry [7], urea online derivatisation via the Ehrlich reaction in a rotor of centrifugal partition chromatography [12], or spot test coupled with diffuse reflectance spectroscopy [13]. The above alternative methods rely either on urea extraction prior to analysis and/or on the use of organic solvents, while they all employ instrumental analysis.

During the last decade, smartphone-based analysis is emerging as an increasingly popular technique, mainly owing to its simplicity, reduced cost, and possibility for in situ analysis [14]. We here present a smartphone-based quantification methodology for cosmetic ingredients. The methodology is simple, fast, and low-cost. Absolutely no extraction step or laboratory analytical instrument is required, which makes the analysis format highly attractive. The proposed methodology which has already been demonstrated for the quantification of glucose in cosmetics [15], is illustrated here for the quantification of urea.

The reaction employed relies on the decrease of methyl red oxidation caused by sodium hypochlorite, in the presence of urea. Methyl red is added exogenously to the emulsion and its decoloration proceeds fast upon subsequent addition of sodium hypochlorite. In the presence of urea, though, decoloration extent is restricted. Hypochlorous acid (and to a lesser extent hypochlorite anion), generated after sodium hypochlorite hydrolysis, reacts with urea to form chloramines [16,17]. Since the generated chloramines are significantly weaker oxidizing agents than hypochlorous acid [18], the presence of urea reduces extent of methyl red decoloration, while the remaining free available chlorine (after consumption towards reaction with urea) is not sufficient to fully bleach the dye. Methyl red decoloration is thus irreversibly proportional to urea concentration. The proposed assay is based on a principle similar to what was described in previous works [8], where, however, urea extraction was required prior to the reaction, while, moreover, dye decoloration was monitored spectrophotometrically.

## 2 Materials and methods

### 2.1 Materials and stock solutions

Commercial bleach was used as a source of sodium hypochlorite. The bleach contained sodium hypochlorite at 4.5 % w/w. Urea 99 % was from Fluka (Geel, Belgium). Colorants utilised were Covarine Yellow WN 1798 (a dispersion of yellow iron oxide in a water/glycerin system), by Sensient Cosmetic Technologies (Crawley, UK), Unicert Blue 05601-J (a disodium salt of triphenylmethane dye) by Sensient Cosmetic Technologies and methyl red, by Merck (Darmstadt, Germany). Methyl red stock at 1.4 mg/mL was prepared in a mixture of 6.8 vol of a 10^−3^ M NaOH aqueous solution with 3.2 vol of ethanol. Unicert Blue 05601-J was dissolved in water at 1.11 mg/mL, while Covarine Yellow WN 1798 was prepared as a 6.47 mg/mL dispersion in water. All cosmetic formulations used are commercial formulations available in the local market and produced by COSMETIC (Athens, Greece). Their composition and certain specifications are provided in Table 1 and in the main text. Standard aqueous buffer solutions from WTW (Weilheim, Germany) were used to calibrate the pH meter.

**Table 1:** Composition, pH, viscosity and transparency of different commercial formulations used in the experiments (source: COSMETIC).

	Cream A	Cream B	Cream C	Shampoo A
**Composition**	Aqua, Glycerin, Caprylic/Capric Triglyceride, Undecane, C14-22 Alcohols, Butyrospermum Parkii Butter, Caprylyl Caprylate/Caprate, Panthenol, Hydroxyethyl Acrylate/Sodium Acryloyldimethyl Taurate Copolymer, Phenoxyethanol, Tridecane, C12-20 Alkyl Glucoside,Perfume, Butylene Glycol, Allantoin, Peg-100 Stearate, Glyceryl Stearate, Xanthan Gum, Citrus Aurantium Dulcis (Orange) Fruit Extract, Benzoic Acid, Tocopherol, Dehydroacetic Acid, Sodium Phytate, Yeast Extract, Helianthus Annuus Seed Oil, Pyrus Malus Fruit Extract, Sodium Benzoate, Potassium Sorbate, Citric Acid, Alcohol	Aqua, Panthenol, Cetearyl Alcohol, Glycerine, Octyl Stearate, Cyclomethicone, Paraffinum Liquidum, Cetearyl, Isononanoate, Dicetyl Phoshate, Ceteth-10 Phoshate, Cetearyl Octanoate, Isopropyl Myristate, Allantoin, Sodium Caproyl/Lauroyl Lactylate, Stearic Acid, Acrylamide/Sodium Acrylate Copolymer, Linallol, Hydroxy Citronellal, Benzyl Alcohol, Eugenol, Imidazolidinyl Urea, Phenoxyethanol, Methyl-/Ethyl-/Propyl-/Butyl-parabens, Perfume	Paraffinum liquidum, Isopropyl myristate, Stearic acid, Octyldodecanol, AMERCHOL L-101, Glycerin, SABOWAX FL 65, Almond oil (Sweet), Triethanolamine, Imidazolidinyl Urea, Methylparaben, Perfume, Propylparaben, BHT	Aqua, Sodium Laureth Sulfate, Tea-Lauryl Sulfate, Cocamidopropyl Betaine, Disodium Cocoamphodiacetate, Bis (C13-15 Alkoxy) Pg Amodimethicone, Salix Alba (Willow) Bark Water, Peg-6 Caprylic/Capric Glycerides, Peg-7 Glyceryl Cocoate, Peg-120 Methyl Glucose Dioleate, Sodium Chloride, Perfume Phenoxyethanol, Propylene Glycol, Citric Acid, Polyquaternium-11, Carbocysteine, Maris Sal (Sea Salt), Disodium Edta, Methylparaben, Glycerin, Panthenol, Propylparaben, Pentylene Glycol, Butylene Glycol, Coumarin, Sodium Benzoate, Ethylparaben, Urtica Dioica (Nettle) Leaf Extract, Panax Ginseng Root Extract, Faex Extract, Santalum Album Seed Oil, Potassium Sorbate
**pH**	4.73	6.51	7.38	5.45
**Transparency**	opaque	opaque	opaque	transparent
**Viscosity, mPa[shear rate]**	∼100 K [1.5 rpm]	∼500 K [0.3 rpm]	13.0 K [3.0 rpm]	∼3.3 Κ [6.0 rpm]

### 2.2 Apparatus

Photometric measurements were conducted on an X-ma 2000 benchtop UV–Vis spectrophotometer (Human Corporation, Seoul, Republic of Korea). For emulsions, pH measurements were performed using a 1:10 dilution of the cosmetic preparation in water, on an InoLab pH Level 1 precision pH meter (WTW GmbH &Co, Weilheim, Germany), equipped with a WTW SenTix 41 combination electrode. No dilution was performed for pH measurement in the low viscosity shampoo. The camera used for picture capturing was a 48-Megapixel camera of a Samsung Galaxy S10 Lite smartphone. Photos were taken in a portable white lightbox (cube of an edge of 23 cm from Shenzhen PULUZ Technology Limited (Shenzhen, China)) equipped with a single row of Light Emitting Diode (LED) light (at 3 cm from the edge and parallel to it) and an opening at the top centre, where the smartphone camera is positioned. A Brookfield (Middleboro, MA, US) DV-E digital viscometer was used for viscosity measurements.

### 2.3 Preparation of emulsion standards and quality control (QC) samples

Standards and quality control (QC) samples of urea in an O/W emulsion or shampoo were prepared by dissolving a suitable amount of solid urea. Standards (between 1 % and 30.2 % w/w urea) and QCs (at 6.03, 10.07, 18.13, 23.32, 27.21 and 30.03 % w/w urea in cream B, or at 2.5 % w/w urea in cream C) were then processed as described below.

### 2.4 Procedure for the quantitative determination of urea in solution

1 mL of a urea stock in water at concentrations ranging between 0 and 13.4 % w/w was mixed with methyl red stock to yield a final methyl red concentration of 22.5 μg/mL. Commercial bleach was then added to reach a final sodium hypochlorite concentration between 0.00 and 0.19 % w/v. Chloramines including nitrogen trichloride are formed upon reaction of urea with sodium hypochlorite which are linked to eye and upper airway irritation [16,17], so the reaction took place in a fume hood. Around 20 min after bleach addition, the absorbance at 420 nm was stabilized and recorded. The experiment was conducted at room temperature (RT) and pH was not fixed.

### 2.5 Procedure for the quantitative determination of urea in a cosmetic formulation

Into 10 g of an O/W emulsion or shampoo containing varying concentrations of urea, a volume of methyl red stock was added and extensively mixed manually to reach a uniform final methyl red concentration of 22.8 μg/g cosmetic formulation. 1 g of the emulsion was then transferred into a 2 mL round vial to which bleach was added and thoroughly mixed manually to a final sodium hypochlorite concentration of 1.51 mg/g cosmetic formulation (in a fume hood). This treated formulation was immediately loaded into the wells of a microstrip (400 μL well volume). Next, the surface of the loaded formulation was flattened with the flat end of a spatula. Standards treatment was in the order of decreasing urea concentration. Within 30 min after sodium hypochlorite addition to the last sample/standard of the processed series, a picture of the series of the colored formulations was captured using a smartphone camera. Even though color intensity in the wells faded significantly with time, the linear relationship between color intensity and urea concentration remained at least 1 h after sodium hypochlorite addition to the first treated sample within the series. The procedure was at RT, while the pH was not fixed.

### 2.6 Procedure for picture capturing and analysis

A 48-Megapixel mobile phone camera and a white lightbox equipped with a single row of LEDs were used to capture pictures of the emulsion/shampoo samples in stable lighting conditions. The camera was positioned above the opening of the lightbox top, lying parallel to and at 23 cm above the microstrip wells loaded with the preparation. Under normal laboratory room lighting, results obtained were unsatisfactory in terms of repeatability and reproducibility. It was also very important that samples and related standards are captured in the same photo for reliable quantification. Additionally, the microstrip wells were not placed right below the light source, to achieve illumination with minimal glare. The parameters of the smartphone camera were: lens aperture: F2.0×, 1× magnification factor, focal length (35 mm): 25.9 mm, automatic adjustment of brightness. The pictures were transferred to a personal computer and were analyzed through the image editing software ImageJ [19] which independently decomposed R(red), G(green) and B(blue) channels. The selected region of interest was typically of an area of 1500–2200 square pixels, around the center of the well to avoid reflection from microstrip material in the edges. Any sub-region within the selected central area which presented a bubble or glare was not considered upon picture analysis. For the specific cosmetic preparations investigated herein, mean RGB intensity or blue channel intensity or saturation channel output were best linearly correlated with urea concentration in the matrix. It is likely that depending on the specific cosmetic matrix under analysis, a different channel value might present the most satisfactory correlation to the concentration of the color-giving compound.

### 2.7 Method validation

Calibration curves were generated in the matrix of the cosmetic formulation, by plotting image analysis channel output versus the nominal concentration of urea in each standard. Linear calibration equation and correlation coefficient (R^2^) were determined. Intermediate Precision, expressed as the percent relative standard deviation of the analytical response (coefficient of variation (CV %)), was assessed by replicate (at least three) measurements of several QC samples under different analysis conditions. The concentration of each QC sample was evaluated using a calibration curve in the same matrix, run together with the QC sample. Accuracy was determined by bias calculation upon standard concentration back-calculation and upon QC concentration measurement.

## 3 Results and discussion

### 3.1 Quantitative method based on digital image analysis

**Fig. 1:**
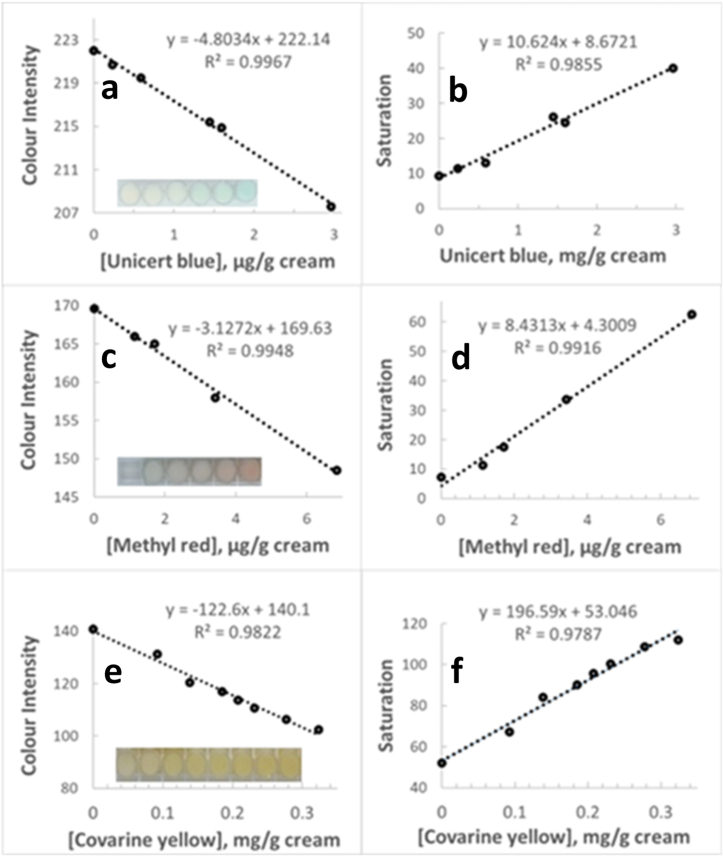
Color intensity (mean of red, blue and green channel values, for Unicert Blue (**a**) and Methyl Red (**c**) and blue component intensity for Covarine Yellow wn 1798 (**e**)) of cream A preparations containing increasing concentrations of a different colorant, plotted against colorant concentration. In the inset, the image of the corresponding wells is given, where colorant concentration is increasing from left to right. In panels (**b**), (**d**), and (**f**) is displayed the saturation profile of the cream A preparations with Unicert Blue, Methyl Red and Covarine Yellow wn 1798, respectively, against colorant concentration. (For interpretation of the references to color in this figure legend, the reader is referred to the Web version of this article.)

An initial proof of concept of a quantitative method based on digital image analysis of a colored topical preparation was attempted using cosmetic preparations containing a range of concentrations of various colorants of blue, red, and yellow color. More specifically, cream A (a commercial O/W cream) preparations containing 0.00–2.96 μg Unicert blue 05601-J/g of cream, 0.00–6.85 μg methyl red/g of cream, and 0.0–320.0 μg Covarine yellow WN 1798 dispersion/g of cream were prepared and loaded into the wells of a microstrip. A picture of the colored formulation was captured using a smartphone camera. When color intensity in the wells was plotted against colorant concentration, a linear relationship was observed (Fig. 1, left). R^2^ ≥ 0.982 was obtained for all colorants. A linear relationship was also observed between color saturation and colorant concentration (R^2^ ≥ 0.978, Fig. 1, right). Cream A ingredients present in the formulation at concentrations above 0.001 % w/w can be seen in Table 1.

These results provide a favourable indication supporting the feasibility of image analysis for the quantification of color-generating ingredients of topical formulations and may open the road for a new, widely applicable analysis format for smartphone-based analysis of topical formulations.

### 3.2 Application of the proposed methodology for urea quantification – optimization of reaction conditions

An application of digital image analysis for ingredient quantification in topical formulations was then attempted: We initially considered quantifying urea concentration in a mock cosmetic formulation to illustrate the validity of the methodology. Quantification relied on the decrease of methyl red decoloration caused by sodium hypochlorite, in the presence of urea.

**Fig. 2:**
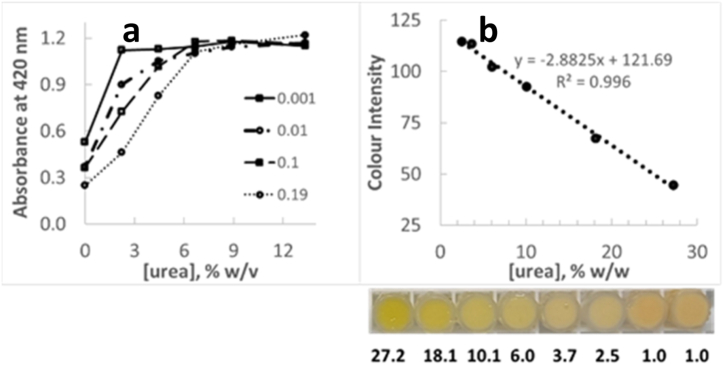
**a.** Dependence of methyl red absorbance at 420 nm (monitored spectrophotometrically) with urea concentration in aqueous solution, in the presence of increasing concentrations of sodium hypochlorite (provided in the legend in % w/w). **b.** Linear relationship between blue color intensity and urea concentration in cream B. Below the graph, an image of the corresponding wells is given, where urea concentration is indicated (in % w/w). (For interpretation of the references to color in this figure legend, the reader is referred to the Web version of this article.)

Urea photometric quantification based on the above principle was first demonstrated in an aqueous solution. An increasing absorbance at 420 nm, where an aqueous non-buffered solution of methyl red absorbs, was measured with increasing urea concentration (between 2.2 and 13.6 % w/w) in the presence of 22.5 μg/mL methyl red and commercial bleach yielding a final sodium hypochlorite concentration between 0.00 and 0.19 % w/v (Fig. 2, left). The initial linear relationship between absorbance at 420 nm and urea concentration was found to extend up to higher urea levels in the presence of higher sodium hypochlorite levels. In the absence of urea, there was no peak at 420 nm. Instead, an intense extra peak at around 320 nm was formed, with a tail extending over 420 nm, thus contributing to blank absorbance at the monitoring wavelength.

Based on the above conclusions, the reaction principle was then attempted inside a mock urea-containing emulsion (“cream B″), where increasing urea concentrations between 0.1 and 30.0 % w/w were contained. The emulsion ingredients present in the formulation at concentrations above 0.001 % w/w can be seen in Table 1, together with certain specifications. Methyl red and sodium hypochlorite were added at a final concentration of 22.8 μg/g cosmetic formulation and 1.51 mg/g cosmetic formulation respectively. Sodium hypochlorite concentration was in the range demonstrated to provide wide linear dependence. Methyl red concentration was such that a measurable color would still remain upon dye decoloration. The resulting colored emulsions were loaded onto microstrip wells. Same as for the experiment in solution, camera-captured color intensity was found proportional to urea concentration in the formulation (up to 30.0 % w/w urea) under the conditions employed (Fig. 2, right). This proportionality was disrupted at urea concentrations below 2.52 % w/w.

The experimental conditions were no further optimized in terms of reagent concentration, since the applied settings were suitable to provide a wide enough linear range for a good majority of cosmetic applications. To investigate the effect of pH on the proposed methodology, emulsion B pH was fixed to 8.5 (a rather extreme pH value for cosmetic applications). A new standard curve was built, with an R^2^ of 0.982 and a linear dynamic range between 2.5 and up to at least 24.2 % w/w urea. This indicates that the effect of pH on the linearity of response is rather minor in terms of goodness of fit. In the following experiments, the pH of the formulations was not adjusted upon urea quantification with the proposed method.

### 3.3 Selection of optimum conditions for smartphone-based measurements

To select the optimum parameters for smartphone-based measurements, calibration graphs were constructed from images of emulsion B, and linear ranges were determined in the Red Green Blue (RGB) and Hue, saturation and brightness (HSB) color spaces, available within the ImageJ application [19]. The analytical parameters calculated are provided in Table 2.

**Table 2:** Analytical parameters for the proposed method registered for various channels of different color models.

Channel	Linear Equation	R^2^	Linear Dynamic Range (% w/w urea)
Red	−0.255x+187.04	0.975	8.1–30.0
Green	−0.452x+171.90	0.966	8.1–30.0
Blue	−3.009x+128.19	0.992	2.5–30.0
RGB average	−1.156x+160.31	0.990	2.5–30.0
Hue	No linear dependence		
Saturation	4.185x+79.49	0.991	2.5–30.0
Brightness	−0.161x+185.96	0.966	2.5–27.2

Based on Table 2 data, very comparable is the linear dependence between measured signal and urea concentration in the cosmetic matrix in terms of goodness of fit and linear dynamic range for the saturation, blue and average RGB channels. Additionally, the slope of the linear dependence is higher in the case of the blue channel and even higher in the case of the saturation channel, which indicates higher sensitivity. Therefore, in the subsequent studies saturation or blue channel output were considered.

### 3.4 Investigation of technical parameters

After linearity and optimum parameters for smartphone-based measurement were established, the proposed methodology was validated in terms of useful analytical range, intermediate precision, and accuracy (Table 3). The coefficient of variation (CV %) on all quality control levels was ≤12.54 % for intermediate precision, indicating marginally acceptable precision. Bias was ≤ ± 4.93 %, indicating good accuracy and a countable matrix effect. In summary, Table 3 data indicate that the methodology is sufficiently reliable for extraction-free urea quantification in the studied formulation.

**Table 3:** Technical parameters of the proposed method for the quantitative determination of urea in cream B formulation.

Intermediate precision		Accuracy			Linearity	
Mean [urea] % w/w	CV % (n)	Measured [urea] % w/w	Spiked [urea] % w/w	Bias %	R^2^	Linear range, % w/w
6.03	12.54 (n = 7)	6.04 (n = 7)	6.03	+0.15	≥0.985 (n = 9)	2.52–30.00
10.07	8.10 (n = 4)	10.57 (n = 4)	10.07	+4.93		
18.13	7.96 (n = 6)	18.58 (n = 6)	18.13	+2.47		
23.32	3.57 (n = 5)	23.27 (n = 4)	23.32	−0.19		
27.21	8.55 (n = 5)	27.11 (n = 5)	27.21	−0.38		
30.03	4.58 (n = 3)	29.15 (n = 3)	30.03	−2.90		

n: number of replicates.

### 3.5 A study of the matrix effect

**Fig. 3:**
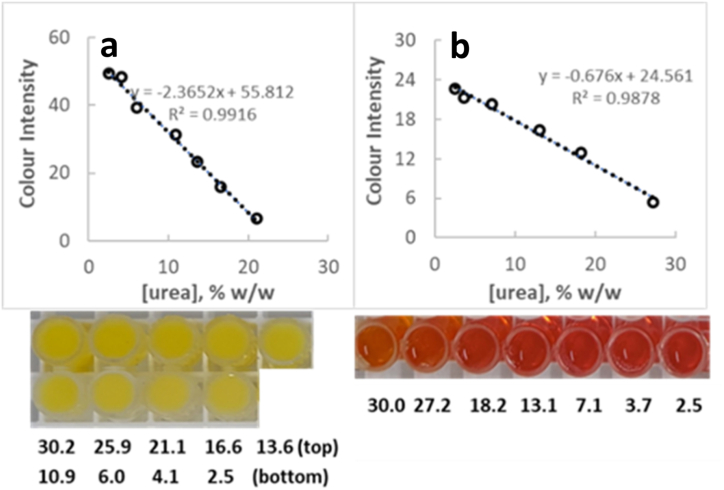
Linear relationship between blue color intensity and urea concentration in cream C (**a**) or in shampoo A (**b**). Below each graph, an image of the corresponding wells is given, where urea concentration is indicated (in % w/w). (For interpretation of the references to color in this figure legend, the reader is referred to the Web version of this article.)

For evaluating recovery and possible non-accountable matrix interferences, two additional commercial cosmetic formulations (a cream (“cream C″-color-free, pH = 7.38) and a shampoo (“shampoo A″- light brown colored, pH = 5.20)), spiked with urea between 2.5 and 30.0 % w/w were prepared. After methyl red and sodium hypochlorite addition at a final concentration of 22.8 μg/g cosmetic formulation and 1.51 mg/g cosmetic formulation respectively, colored emulsions were loaded onto microstrip wells and standard curves were built (two replicates). The ingredients present in the formulations at concentrations above 0.001 % w/w, together with their pH, transparency and viscosity, can be seen in Table 1. Calibration curves showed good linearity and acceptable results of the back-calculated concentrations over the range of 2.5–21.1 % w/w urea for cream C and 2.5–27.2 % w/w urea for shampoo A (Fig. 3). For all standards, standard concentration was back-calculated within ±10.2 % of their nominal concentration (+34.1 % close to the lower limit of linearity (2.5 % w/w)). This experiment indicates the countable effect of the formulation matrix on the linearity of the proposed analysis method even in the presence of a slightly colored formulation, as in shampoo A. It should be noted that linearity remained (R^2^ = 0.986) even where the wells were colored red, as in shampoo A. Red coloration occurred, despite the pH of 5.45, due to the shift in the transition interval of the indicator because of methyl red interaction with sodium dodecyl sulfate [20], an ingredient of shampoo A. Similarly, when fixing another emulsion's pH to a value where methyl red is colored orange, smartphone-captured color intensity was still proportional to urea concentration in the formulation (R^2^ = 0.991, data not shown). Concluding, the effect of pH and/or viscosity and/or transparency of the formula on the goodness of fit to the linear model appears little significant within the width of pH and viscosity values commonly encountered in a good majority of cosmetic formulations (pH 5.4–7.5; viscosity 3k-100k mPa). Their effect on the linear dynamic range is more important, however the linear range is still appropriate for analysis of a very large fraction of cosmetic matrices, containing medium levels of urea. The narrower linearity range observed may be the result of the different background in the two matrices. This background may lead to saturated pixels in the picture of the wells at different levels of remaining methyl red dye, i.e at different levels of urea. Saturated pixels are very likely to lead to unreliable quantification results, i.e. may narrow down linearity range.

### 3.6 Accuracy upon analysis of commercial products

To further demonstrate the validity of the approach for quantification of urea within quality control in the dermatological and cosmetic industry, we analyzed three mock urea-containing emulsion preparations (QCs) based on “Cream C″ or “Cream B”. Urea quantification therein was attempted by the standard addition method, where aliquots of the analyzed sample are spiked with different concentrations of the analyte of interest. Calculation of unknown analyte concentration by the standard addition approach relies on extrapolating the zero-analyte signal. Since, however, the linearity of the method employed does not expand to urea concentrations <2.5 % w/w, such extrapolation is not valid. For this reason, we employed extrapolation to higher analyte concentrations and a calibration standard containing urea in the corresponding cosmetic matrix at a concentration >2.5 % w/w was used. Analysis results by the standard addition method, are given in Table 4, together with the accuracy of determination. Bias in quantification was in all cases ≤ ±10.9 %, (average of at least two replicate experiments with a CV ≤ 13.6 % in all cases) which indicates the validity of the approach.

**Table 4:** Accuracy of the proposed method for the quantitative determination of urea in commercial formulations.

Product	Accuracy		
Name	Meas. [urea], % w/w	Spiked [urea], % w/w	Bias %
Cream C	2.26	2.50	−9.72 (n = 3, CV = 13.6 %)
Cream B (pH 6.5)	11.09	10.0	+10.91 (n = 2, CV = 4.95 %)
Cream B (+ammonia)	9.99	10.0	−0.04 (n = 2, CV = 6.67 %)

### 3.7 Inteferences

In one of the preparations in cream B, ammonia was added (pH was raised to 8.5). Ammonia, as well as other nitrogenous compounds that are likely to be present in a cosmetic preparation (such as amino acids, proteins), or phenols and carbohydrates might also consume free available chlorine [16], reducing extent of decoloration. Table 4 data show that even in the presence of ammonia, the proposed analysis setup in combination with a standard addition methodology was able to accurately determine urea concentration. All together, these results demonstrate successful accounting of matrix effects by the proposed methodology to achieve accurate, smartphone-enabled determination of urea in various cosmetic formulations.

Other oxidizing compounds potentially present in certain commercial bleach preparations, such as hydrogen peroxide, should also contribute to dye decoloration, in addition to hypochlorite salts. This contribution, not following the path via hypochlorous acid, is not expected to be modified by the presence of urea. Hence, the presence of such oxidizing agents would generate a stable, urea-independent, additional background decoloration. Although the relative concentration of the dye in the proposed method might have to be adjusted (to account for the higher oxidizing capacity of the bleach), application of the standard addition method should still allow urea quantification, taking background oxidation into account.

### 3.8 Comparison of analytical performance with literature methods

A comparison of the analytical performance of the proposed method versus literature methods for urea quantification for quality control purposes in the dermatological and cosmetic industry is provided in Table 5. A direct comparison of analytical figures of merit of Table 5 between the proposed method and literature methods is not feasible in all cases. This is since Pearson's coefficient of linearity, LOQ and precision (repeatability or reproducibility) were determined in most reported studies using urea standards in solution, and not in the cosmetic matrix, as in the current work. Upon comparison of those figures of merit determined in working samples (given in italics in Table 5) it becomes clear that the proposed method is inferior to chromatographic methods in terms of precision, while it is comparable to half of the methods or inferior to the other half, in terms of accuracy.

**Table 5:** Comparison of the analytical performance of different literature methods for urea quantification in dermatological and cosmetic formulations.

Analytical method	Need for extraction step or use of organic solvent	Linearity (R^2^)	Repeatability or Reproducibility CV (%)	Accuracy (bias %)	LOQ	Source
Spectrophotometry	YES	>0.999 (solution)	2.72 (solution)	≤ ± *1.00 (ointment*)	7.63 μg/mL (solution)	[7]
HPTLC-densitometry	YES	>0.99 (solution)	3.84 (solution)	≤ ± *3.73 (ointment)*	2.77 μg/mL (solution)	[7]
Potentiometry (pH–enzyme electrode)	YES				*at least 0.*6 mg *urea/g formulation (ointment*)	[9]
Kjeldahl (official method)	harsh process			≤ ± *11.0 (emulsion)*		[13]
Hydrophylic HPLC -UV detection	YES	>0.997 (solution)	≤ *1.53 (emulsion)*	≤ ± *3.22 (lotion)* ≤ ± *3.13 (conditioner)* ≤ ± *2.34 (gel)*	30 μg/mL (solution)	[10]
Hydrophylic nanoHPLC- UV detection	YES	0.999 (solution)	≤2.80 (solution)	≤ ± *4.44 (emulsion)*	50 μg/mL (solution)	[11]
Centrifugal partition chromatography	YES	0.998 (solution)	*0.7 (emulsion)*			[12]
Diffuse Reflectance Spectroscopy	YES	0.994 (solution)	≤1.90 (solution)	≤ ± *12.0 (emulsion)*	21.65 μg/mL (solution)	[13]
Digital image colorimetry (smartphone)	NO	≥ *0.985 (emulsion)*	≤ *12.54 (emulsion)*	≤ ± *10.91 (emulsion)*	*25.*2 mg *urea/g formulation (emulsion, shampoo)*	this work

The non-necessity for laboratory analytical equipment, organic solvents or lengthy pre-treatment procedures are important advantages of the proposed methodology that should render it the method of choice in those settings where the determined analytical figures of merit are sufficient for the analysis purpose. Although azo dyes such as methyl red, are water pollutants that may promote toxicity, mutagenicity and carcinogenicity [21], the procedure described does not generate liquid waste which allows easier waste management.

## 4 Conclusions

We here report a simple, extraction-free, smartphone-enabled methodology for the quantification of urea in cosmetic formulations. The validity and robustness of the methodology was demonstrated by determining its favourable technical characteristics in a W/O emulsion and by quantifying urea with acceptable accuracy in commercial preparations of different viscosity, transparency, pH, and/or color, even in the presence of interfering ammonia. The standard addition method was successfully employed for quantification purposes to account for matrix effects and provide an acceptably accurate measurement of urea content, over a concentration range relevant to cosmetic applications.

Abolishing the need for analyte extraction, for use of organic solvents and for analytical instrument-based quantification in cosmetics analysis are great advantages, reducing significantly the cost, environmental impact, length, and complexity of analysis. The width of applicability of the approach to quantify further cosmetic, galenic, or even food ingredients in emulsions, capable of generating a colorful reaction product within the formulation remains to be studied.

## Funding

This research did not receive any specific grant from funding agencies in the public, commercial, or not-for-profit sectors.

## CRediT authorship contribution statement

**Georgia Eleni Tsotsou:** Writing – review & editing, Writing – original draft, Validation, Methodology, Investigation, Formal analysis, Conceptualization.

## Declaration of competing interest

The authors declare no competing interests.
